# Recent progress of second near-infrared (NIR-II) fluorescence microscopy in bioimaging

**DOI:** 10.3389/fphys.2023.1126805

**Published:** 2023-02-21

**Authors:** Tian Wang, Yingying Chen, Bo Wang, Mingfu Wu

**Affiliations:** Department of Obstetrics and Gynecology, Tongji Hospital, Tongji Medical College, Huazhong University of Science and Technology, Wuhan, China

**Keywords:** second near-infrared (NIR-II) fluorescence, *in vivo* bioimaging, fluorescent probe, confocal microscopy, light-sheet fluorescence microscopy, wide-field microscopy

## Abstract

Visualizing biological tissues *in vivo* at a cellular or subcellular resolution to explore molecular signaling and cell behaviors is a crucial direction for research into biological processes. *In vivo* imaging can provide quantitative and dynamic visualization/mapping in biology and immunology. New microscopy techniques combined with near-infrared region fluorophores provide additional avenues for further progress *in vivo* bioimaging. Based on the development of chemical materials and physical optoelectronics, new NIR-II microscopy techniques are emerging, such as confocal and multiphoton microscopy, light-sheet fluorescence microscopy (LSFM), and wide-field microscopy. In this review, we introduce the characteristics of *in vivo* imaging using NIR-II fluorescence microscopy. We also cover the recent advances in NIR-II fluorescence microscopy techniques in bioimaging and the potential for overcoming current challenges.

## Introduction

Fluorescence imaging has been widely used in biomedicine due to its advantages of high sensitivity, non-invasiveness, and lack of radiation hazard (Schouw et al., 2021). Fluorescence imaging uses endogenous fluorescence (spontaneous fluorescence) or fluorescence stimulated by an external labeled probe (induced fluorescence) to acquire images. This process includes three key steps: signal excitation, collection, and detection. When the fluorescent probe binds to cell or tissue, a light source with a specific wavelength is used to irradiate the marker, which causes emission through excitation of the fluorophore. At this point, spontaneous fluorescence often becomes the background of the image. Then, through collecting, splitting, filtering, and focusing the emitting light path, the excited fluorescence signal enters the photodetector to complete the photoelectric conversion. This is followed by signal amplification, analysis, and processing, to finally obtain the image (Meng et al., 2022). As a promising imaging method, fluorescence imaging has the advantages of high temporal and spatial resolution and few side effects, which cannot be achieved by *in vitro* imaging at the cell and tissue level.

Traditional biological imaging techniques use visible light (400–700 nm) and NIR-I (the first near-infrared region, 700–900 nm), which have the following limitations (Duan and Yang, 2022). First, due to the influence of absorption and scattering when light propagates in biological tissues, the imaging depth and signal-to-background ratio (SBR) are not ideal. The objects to be visualized are usually limited to cell and tissue samples with low thickness. Second, spontaneous fluorescence exists in biological tissues, which often forms the background, interfering with imaging and causing a decrease in the SBR and clarity. Third, the excitation wavelength of visible light and NIR-I fluorescence is shorter, the photon energy is higher, and the safety threshold of excitation light is lower. The absorption of excessively strong excitation light by biological tissues will lead to tissue damage. Compared with visible light and NIR-I fluorescence imaging, NIR-II (the second near-infrared region, 1000–1700 nm) fluorescence imaging has the obvious advantages of imaging clarity and laser power safety *in vivo* due to the longer wavelength used.

In recent years, the NIR-II fluorescence microscopy technique has achieved a breakthrough in terms of high temporal resolution, high spatial resolution, high SBR, and strong penetrability of deep tissue, which can be attributed to the development and improvement of fluorescence probes and imaging instruments. Most NIR fluorescence imaging is based on confocal microscopy. However, the point scanning property of confocal microscopy limits the imaging of dynamic samples to a small volume or a series of fixed samples at a certain stage of a process. Recently, a variety of microscopy techniques for wide-field acquisition and excellent spatial and temporal resolution have been developed, such as confocal microscopy, light-sheet fluorescence microscopy, and wide field microscopy techniques (Zhang et al., 2022a). For large-volume imaging *in vivo*, beam-shaping strategies are widely used to optimize microscopy techniques. In this review, we introduce the characteristics of *in vivo* imaging *via* NIR-II fluorescence imaging and review studies of NIR-II fluorescence microscopy technology to provide support for basic research and clinical applications of NIR-II fluorescence microscopy.

### NIR-II fluorescent *in vivo* bioimaging

Because the energy of photons of wavelength over 900 nm is lower than the bandgap energy of conventional Si-based semiconductor materials, conventional silicon-based sensors are transparent to photons in the long wavelength of the NIR. NIR-II detection generally uses indium gallium arsenide (InGaAs) sensors with high quantum efficiency in the range of 900–1700 nm, with wide application in industrial detection, military equipment, security, and other fields (Potma et al., 2021).

With the improvement of detector performance and the development of new fluorescent probes, *in vivo* NIR-II fluorescence imaging has become a research hotspot (Li et al., 2022a; Baghdasaryan et al., 2022; Niu et al., 2022; Xu et al., 2022; Liu et al., 2023). The first *in-vivo* NIR-II fluorescence imaging was achieved by Hongjie Dai in 2009 (Welsher et al., 2009). They visualized the inherently near-infrared luminescence of mice by insulating low-dose “swapped” single-walled carbon nanotubes (SWNT). The InGaAs camera captured the image in the wavelength range of 1000–1700 nm, clearly visualized the circulation of single-walled sodium carbon tubes in the subcutaneous vasculature, and detected higher fluorescence strength in the liver and spleen of mice, which is due to the tendency of nanostructured materials to accumulate in the organs of the reticuloendothelial system. In addition, Bawendi et al. (Bruns et al., 2017). Have reported a class of high-quality short-wave infrared emitting indium arsenide quantum dots that can be used for clear full-body imaging of mice and measurement of the heart rate and breathing rate of resting mice without contact. Zhen Cheng et al. (Suo et al., 2019; Zheng et al., 2021). Developed new NIR-II molecular imaging probes for early cancer detection, metabolism studies, and treatment. Chen Xiaoyuan et al. (Wang et al., 2019a; Tian et al., 2020) developed a precision diagnosis and treatment system based on the precise detection of NIR-II fluorescence imaging and multiple NIR-II *in vivo* imaging. Fan Zhang et al. (Liu et al., 2018; Wang et al., 2021) developed erbium-doped (Er3+) rare earth near-infrared probes for diagnosis, navigation surgery, and lifetime imaging applications using NIR-II with high SBR. Wang Qiangbin et al. (Yang et al., 2021a; Yang et al., 2021b) carried out *in situ*, real-time, high-sensitivity, and high-signal-to-noise (SNR) imaging of living tissue by using NIR-II fluorescence imaging technology with silver containing low-toxicity quantum dots. Hong Xuechuan et al. (Zheng et al., 2020; Zhou et al., 2020) carried out small-molecule drug-related biological imaging of blood vessels and tumors for early diagnosis of diseases and drug development. Fan Quli et al. (Tang et al., 2018; Li et al., 2019) developed a series of semiconductor polymers with the emission properties of NIR-II as well as the use of multi-mode imaging NIR-II organic fluorescent probes for the photothermal treatment of focal diseases and other applications. The rapid development of NIR-II *in vivo* imaging technology also provides a good opportunity for clinical precision diagnosis and treatment. In 2019, Cheng Zhen et al. (Suo et al., 2019; Hu et al., 2020) reported the application of NIR-II fluorescent endoscopy for the targeted imaging of colorectal cancer. They also performed human liver cancer surgery under the guidance of multi-window fluorescence imaging of visible light, NIR-I, and NIR-II for the first time, ushering in a new era of clinical application of NIR-II. The application of NIR-II fluorescence imaging technology has evolved from cells to the diagnosis of diseases in large animals and even humans. Although there have been some preclinical studies of NIR-II fluorescence imaging, many challenges still need to be addressed before it can be used in the clinic.

### NIR-II fluorophores

The development and production of a number of functional near-infrared fluorophores for highly specific anatomical and molecular imaging is conducive to the application of NIR-II fluorescence imaging technology in basic research and preclinical practice, and effectively promotes the development of this field. From a clinical perspective, the NIR fluorophore should have certain characteristics, including high safety, high stability, high quantum yield, low toxicity, minimal or no accumulation in non-target organs, and long emission wavelengths. A variety of NIR fluorophores have been developed for *in vivo* fluorescence imaging, including inorganic fluorophores (such as carbon nanotubes, quantum dots, lanthanide-doped nanoparticles) (Zhong et al., 2017; Chen et al., 2018; Zhang et al., 2020; Qin et al., 2022a; Teng et al., 2022) organic fluorophores (such as organic small molecules, polymers and activatable fluorescence probes) (Antaris et al., 2016; Li et al., 2022b; Zhang et al., 2022b; Gao and Lei, 2022; Zhao et al., 2022) A variety of inorganic materials have been used in fluorescent imaging, this may cause safety concerns about cumulative toxicity. Considering the unknown long-term toxicity, these inorganic nanomaterials are difficult to translate to the clinic. Organic fluorescent probes has adjustable physical and optical properties controlled by structural engineering, processability and good biocompatibility, therefore presents a better alternative (Table 1) (Zhu et al., 2020; Cui et al., 2022).

**TABLE 1:** The summary of advantages and disadvantages of NIR-II fluorophores.

NIR-II fluorophores	Advantages	Disadvantages
Inorganic		
carbon nanotubes	good photostability	low quantum yield
		high excitation intensity
quantum dots	narrow emission wavelength	toxicity
	broad excitation wavelength, superior quantum yield	
	long fluorescence lifetime	
lanthanide-doped nanoparticles	high thermal and chemical stability	low quantum yield
	high imaging resolution	unknown long-term toxicity
	high penetration depth	limited spatial imaging resolution
	no photobleaching	long collection time
Organic		
organic small molecules	high biocompatibility	low quantum yield
	fast excretion	short emission wavelength
	superior optical properties	time-consuming synthesis and purification process
polymers	superior optical properties	limitation of biocompatibility slow excretion
	adjustable structure	
activatable probes	more accurate for specific disease diagnoses	low quantum yield
	high resolution	low photochemical stability
	fast response	

Organic compounds based on small molecules show strong prospects for clinical transformation due to their low toxicity, high synthetic repeatability, and simple chemical modification. Small molecules are metabolized by the body without the production of additional metal ions. Dai et al. (Zhou et al., 2022) introduced a water-soluble nanoparticle fluorescent molecular probe with a donor-receptor-donor (D-A-D) skeleton, which showed good photostability and pharmacokinetics, pioneering the development of nanoparticle small-molecule fluorescent probes. Although small-molecule fluorophores have made great strides in NIR-II fluorescence imaging, most fluorophores are only used in small animals, and are still a long way from clinical application. Fortunately, scientists (Han et al., 2022; Kasprzyk et al., 2022; Rodriguez-Luna et al., 2022) have successfully applied some small molecular fluorophores to large animals (rabbits and monkeys) and humans to accelerate their clinical translation. However, ICG has low photostability and its fluorescence is easily quenched (Potharazu and Gangemi, 2022). Using an aggregation-induced emission (AIE) probe, Tang et al. (Qi et al., 2018a) performed high-contrast NIR-II bioimaging of cyborg monkey arteries. These amazing achievements provide compelling evidence for future clinical translational research. Tian et al. (Hu et al., 2020) recently guided the surgical resection of 23 human primary and metastatic hepatocellular carcinomas using fluorescence imaging in combination with ICG, which was the first clinical application of the technology.

In addition, activatable organic NIR-II fluorescent probes generate fluorescent signals in the living system only after responding to the target analyte, thereby displaying a higher SBR. Thus, they have attracted increased attention for biomedical and clinical research (Zhang et al., 2022b). Activatable organic NIR-II fluorescence probes have been used in the detection of reactive oxygen species (ROS), reactive nitrogen species (RNS), reactive sulfur species (RSS), pH, viscosity and enzyme. This advanced, non-invasive and highly specific optical imaging mode shows promising prospects in exploring the pathophysiology of diseases and potential clinical translation. Ren et al. designed a novel class of polymethine dyes (NIRII-RTs) with bright (quantum yield up to 2.03%), stable and anti-solvent quenching NIR-II emission, together with large Stokes shifts, realizing the real-time monitoring of drug-induced hepatotoxicity (Ren et al., 2021). Qin et al., designed a novel dye scaffold NIRII-HDs, realizing reliable NIR-II imaging of different diseases in mouse models and evaluation of the redox potential during a liver injury *in vivo* with high fidelity (Qin et al., 2022b). He et al. constructed NIR-II Cy3s (a series of stable and multifunctional NIR-II dyes) for reversible monitoring of HClO/RSS-mediated redox processes in the pathophysiology environment (He et al., 2022). Although several examples of NIR-II fluorescence imaging have been used in preclinical studies, there are many challenges that need to be addressed before it can be used in a clinical setting.

### NIR-II fluorescence microscopy techniques

New microscopy techniques have facilitated many key advances in biology. For example, microscopy is useful for observing the transfer of the human immunodeficiency virus (HIV) (Hubner et al., 2009) from cell to cell, the separation of individual chromatids during cell division, the transient activity of the entire brain of zebrafish larvae, and the neuronal activity of entire worms (Gao et al., 2012; Hontani et al., 2022; Ishii et al., 2022). *In vivo* imaging studies through macro NIR-II imaging can not only detect circulation in the aorta and tiny blood vessels, but also be used for the imaging of various organs, such as the heart (Greiner et al., 2022), liver (Ojha et al., 2022), lung (Mirsanaye et al., 2022), kidney (Sonoda et al., 2022) and intestine (Hanafy et al., 2022). However, the visualization of tissue microstructure requires imaging systems with larger magnification for improved spatial resolution and contrast of biological tissue and clear imaging of biological microstructure.

### Confocal and multiphoton microscopy

**FIGURE 1:**
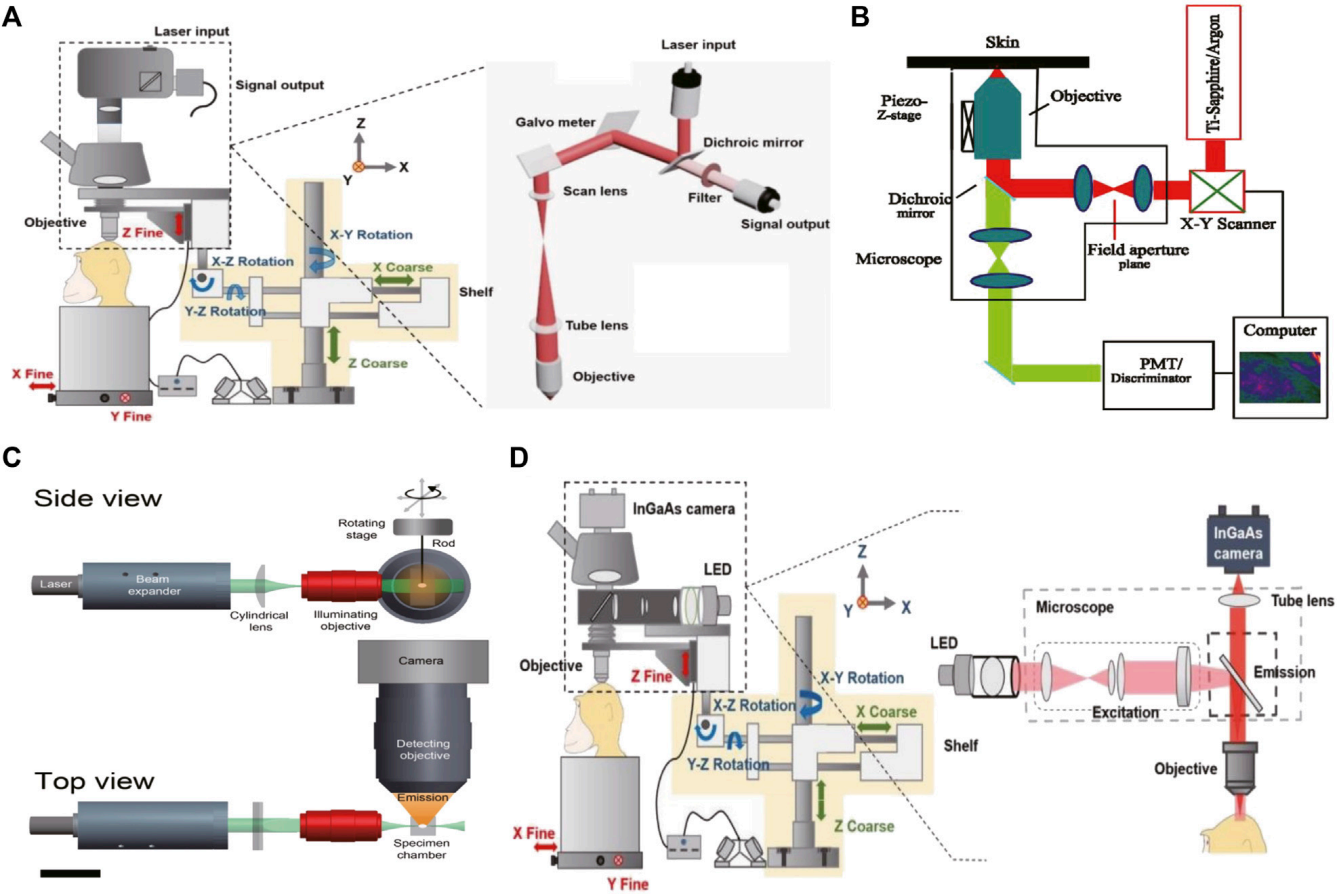
The schematic illustration of the NIR-II fluorescence microscopic imaging system **(A)**. Schematic illustration of the confocal fluorescence microscopic imaging system. Copyright Year 2020, Theranostics (Cai et al., 2020) **(B)**. Schematic illustration of the multiphoton fluorescence microscopic imaging system. Copyright Year 2001, Opt Express (Masters and So, 2001) **(C)**. Schematic illustration of the light sheet fluorescence microscopic imaging system. Copyright Year 2011, J Histochem Cytochem (Santi, 2011) **(D)**. Schematic illustration of the wide-field fluorescence microscopic imaging system. Copyright Year 2020, Theranostics (Cai et al., 2020).

So far, confocal microscopy and its non-linear counterpart multiphoton microscopy have been considered the gold standard techniques for fluorescence bioimaging (Ramani et al., 2022; Teo et al., 2022). Confocal microscopes can eliminate out-of-focus signals by placing a pinhole aperture at the conjugate point of the focus of the detecting objective lens and the focus of the concentrator. The pinhole ensures that only the signal from the focal point in the sample can pass through and be collected by the detector. Point by point scanning yields a three-dimensional (3D) image without fuzzy signals. Due to this point-to-point principle, this method can provide high-contrast optical slice images of considerable depth in thick scattered samples (Figure 1A) (Cai et al., 2020). However, point scanning characteristics also limit the imaging speed of large samples. The exposure of fluorescence bioimaging can cause additional damage, such as photobleaching of the fluorophore and photon toxicity. Therefore, confocal microscopy is the best choice for small volume imaging (Paganelli et al., 2022).

Multiphoton microscopy is an extension of confocal microscopy (Figure 1B) (Masters and So, 2001; Cai et al., 2020; Yang et al., 2022), but does not exclude pinholes. Based on the non-linear multi-photon excitation principle, excitation only occurs at the maximum intensity point of the illumination, which can effectively attenuate the defocus signal. This special application of the non-linear multiphoton excitation principle gives it almost the same effect as a pinhole. In addition to high contrast and high SBR, there are several other advantages. First, the large separation between the excitation spectrum and emission spectrum eliminates the mixed fuzzy signal. Another is the use of longer wavelengths, especially in the NIR-II or longer spectral window, which is beneficial for deep *in vivo* bioimaging as discussed earlier. However, multiphoton excitation requires very expensive ultrashort pulsed lasers. In addition, it limits the choice of available excitation wavelengths, and thus the choice of fluorophore (Akbari et al., 2022).

Qian Jun et al. (Chu et al., 2014; Alifu et al., 2018; He et al., 2020; Feng et al., 2021) built a set of near infrared laser scanning confocal microimaging systems (NER-LSCM) based on the commercial Olympus FV1000 laser scanning confocal microscope, including a near-infrared laser source (785 nm), optical path climbing system, Olympus FV1000 laser scanning confocal microscope, dichroic mirror, fiber coupler, optical fiber, near infrared response PMT, signal amplifier, data acquisition card, and computer. The researchers injected 300 µl of PBS dispersion of ICG nanoparticles at a concentration of 500 µM into the caudal veins of anesthetized 8-week-old ICR female mice, and then fixed the ears or brains of mice under the NIR-LSCM system for fluorescence imaging to obtain a 3D reconstruction of the blood vessels in the ears and brain of the mice. They also coated TQ-BPN, an organic dye with aggregation-induced luminescence effects, to obtain stable TQ-BPN nanoparticles with good bioaffinity. They then combined them with NIR-LSCM for deep-tissue fluorescence imaging in living mice, achieving a depth of 700 µm for cerebral vascular imaging. The researchers thus surpassed this limitation of laser scanning confocal microscopy in cell imaging and thin tissue imaging, making the application of laser scanning confocal microscopy in deep fluorescence imaging of living tissue a reality. The team also built a new two-photon excited near-infrared fluorescence microscopic imaging system. With the help of fluorescent probe TQ-BPN nanoparticles and excitation with the 1040 nm fs laser located in the NIR-II region (1000–1400 nm), the near-infrared photomultiplier tube (H7422-50) was used as the fluorescence detector to detect the two-photon near-infrared fluorescence signal located in the NIR-I region (700–900 µm), and achieve large-depth fluorescence microscopic imaging of 950 µm in the brain blood vessels of living mice.

Hongjie Dai et al. (Antaris et al., 2017; Zhang et al., 2018) used 808 nm epi-fluorescence excitation to build an amplification and focusing optical system composed of two achromatic lenses. Through the intact skin and skull, non-invasive fluorescence imaging of cerebral blood vessels of mice at 1300–1400 nm was achieved in this window, and the mouse cerebrovascular system was analyzed at a depth of more than 2 mm. At a clinical level, the middle cerebral artery embolization model can accurately distinguish the changes in vascular morphology and structure, and perform feature recognition and boundary detection of malignant tumors in the brain: these applications all require a microscopic imaging system with higher magnification to achieve accurate analysis. The realization of large-depth microscopic imaging of living biological tissues indicates that confocal NIR-II microscopy holds great promise in the field of biomedicine.

### Light-sheet microscopy (LSM)

Because of the 3D volume imaging ability, low phototoxicity, low background, and fast sectioning speed, light microscopy has attracted great attention in biological and medical research (Figure 1C) (Santi, 2011; Cai et al., 2020; Patel et al., 2022; Pesce et al., 2022; Ma et al., 2021). Efforts to improve resolution, volumetric imaging rates and tissue penetration depth have been ongoing for decades. Scanning structured lighting and single molecule localization give LSM limited subdivision resolution. The penetration depth can be effectively increased by means of two-photon excitation and self-reconstructing Bessel or Airy beams. Recently, it was reported that the excitation and emission of NIR-II LSM has been extended to 1320 nm and 1700 nm, respectively, which inhibits the scattering of excited and emitted light. The NIR-II LSM enables optical section and volumetric imaging to penetrate up to 750 μm through the intact scalp and skull of mice. However, due to diffraction limitations on the long wavelength of the detected light, the resolution of the NIR-II LSM with a typical planar plate using a cylindrical lens is approximately two to three times lower than that of the visible-light LSM. Although the NIR-II LSM has deeper penetration than the visible LSM, it still experiences light scattering effects, resulting in feature smearing and undesired background.

The excitation light is a thin piece of light, and the signal of the excitation layer is collected by the vertical arrangement of the detection light path and the illumination light path. LSM changes the way that light excitation and collection is performed. Light is modeled into a plane through a cylindrical lens, and the entire plane is required to pass through the sample. This results in stringent sample requirements and the need for optical transparency in general. Therefore, it is a big challenge to realize cerebrovascular imaging with LSM. Some advanced beams, such as Bessel and Airy beams, have also been introduced in light-Sheet fluorescence microscopy (LSFM) (Adams et al., 2015; Amich et al., 2020). Compared to the conventional focused Gaussian beams that solve the beam divergence problem, the non-diffraction properties of these two advanced beams can contribute smaller core sizes over extended longitudinal ranges. However, LSFM-based Bessel or Airy beams must be solved with other techniques to overcome the sidelobe negative effects. Returning to the principle of multiphoton excitation, because the emission profile of multiphoton excited fluorescence is proportional to the exponent of illumination intensity, the sidelobe of the Bessel/Airy sheet is greatly suppressed and exhibits excellent axial resolution and penetration depth due to the longer wavelength used (Hobson et al., 2022).

To produce thin sheets of light, so-called lattice photomicroscopes (LLSM) use two-dimensional optical lattices of Bessel beams to achieve near diffraction limit resolution in X, Y, and Z directions with high SNR. This avoids the need for excessive excitation light in creating high-contrast images, resulting in low photobleaching/phototoxicity, while maintaining fast acquisition times. LLSM has proven to be a valuable tool for dynamic bioimaging in a variety of sample types (Tsai et al., 2020). There is a trade-off between sample size/maximum imaging volume and achievable spatial resolution for a particular optical piece technology. Although LLSM has the spatial resolution, it has the smallest imaging volume of nearly 2.5 × 10^5^ m^3^ (i.e., very few cells are cultured). In contrast, IsoView imaging has a spatial resolution of only more than half the spatial resolution, but its imaging volume is close to 1.28 × 10^8^ m^3^ (i.e., the entire *Drosophila* embryo).

With the development of NIR fluorescence bioimaging technology, the combination of LSFM and near infrared fluorophores has been explored, with great potential. More recently, Dai et al. (Wang et al., 2019b) developed near-infrared II (NIR-II) (1000–1700 nm) structured-illumination light-sheet microscopy (NIR-II SIM). The wavelength of light excitation and emission is as high as ∼ 1540 nm and ∼1700 nm, respectively. In a biological demonstration, the combined microscopy technique achieved non-invasive *in vivo* imaging of the living mouse brain at a depth of 2 mm, with an axial resolution of less than 10 μm, resulting in dynamic processes such as 3D molecular imaging of highly abnormal tumor microcirculation and important immune checkpoint proteins, and single-cell scale programmed death ligand one receptors in tumors. NIR-II SIM provides an additional tool for non-invasive volumetric molecular imaging of immune cells in living mammals.

### Wide-field microscopy

Compared with the previous two techniques, wide-field microscopy offers higher spatial and temporal resolution and is easier to operate (Figure 1D) (Cai et al., 2020; Jiang et al., 2020). First, the contrast scanning point detector needs external *X*-axis and *Y*-axis scanning devices for surface detection to produce two-dimensional images at a time, such as an image of 640 *512 pixels, so that the time resolution is higher. Second, because there is no need for beam focusing and point excitation, the requirements for the beam are significantly reduced, and the operation is simple. Therefore, although the background suppression of wide-field microscopy is inferior to that of confocal microscopy and LSM, it is conducive to popularization and application because of its significantly lower requirements for samples and experimentalists (Table 2) (Lee et al., 2020). In order to improve the effect of background suppression of NIR-II, Qian Jun, et al. (Feng et al., 2019) carried out fluorescent wide-field microscopic brain imaging in the NIR-IIx region, proving that this band has excellent optical section ability. In the 1400–1500 nm window, water absorption of light greatly inhibits the background. Therefore, microcapillary tubes with a diameter of only 4.1 μm at 900 µm can be distinguished, and even vessels at 1.3 mm can be identified. They also develop a new nanoparticle fluorescence positioning microscopic imaging system, which can enable wide-field excitation, array detection, deep imaging depth, high temporal resolution, good spatial resolution, easy operation, and other advantages, and can achieve high-power detection of deep tissue. So far, it has been applied in targeted chemotherapy of cervical cancer and cerebrovascular research in mice.

**TABLE 2:** The summary of advantages and disadvantages of NIR-II fluorescence microscopies.

NIR-II fluorescence microscopy	Advantages	Disadvantages
Confocal and Multiphoton Microscopy		
	high contrast	low imaging speed, photobleaching
	high SBR	photon toxicity
	eliminating out-of-focus signals	expensive excitation source
Light-Sheet Microscopy		
	low phototoxicity	limited resolution
	low background	limited volumetric imaging rates
	fast sectioning speed	limited tissue penetration depth
Wide-Field Microscopy		
	higher temporal and spatial resolution	weak background suppression
	precise location of drug distribution	lacking advanced detectors
	deep imaging depth	
	commercially available	

Bawendi, et al. (Bruns et al., 2017; Qi et al., 2018b) introduced a class of indium arsenide quantum dots. The dynamic imaging of blood flow in healthy tissues and tumor edges was carried out using a NIR-II fluorescence wide field microscopic system, thus generating z section images of normal and abnormal vascular systems in mice. Irregular blood vessels and oscillating “pendulum” blood flow were observed at tumor edges. The healthy hemisphere showed a normal network of blood vessels and regular blood flow. Relevant data showed that the NIR-II fluorescence wide field microscopic system could not only visualize the rich capillary structure of mice but also measure blood flow velocity with high time resolution.

In addition, the NIR-II fluorescence wide field microscopy technique can achieve the visualization of tissue vessels with high spatial and temporal resolution. Tang Benzhong et al. (Qi et al., 2018b) developed new near-infrared aggregation-induced emission (AIE) nanoparticles. With the help of the NIR-II fluorescence wide-field microscopic imaging system, the process of photo-thrombotic ischemia (PTI) and blood-brain barrier (BBB) injury in the brains of mice was accurately monitored. In addition to small photogenic thrombus, the fluorescence wide-field microscopy system can also accurately detect the formation of large thrombus after middle cerebral artery Occlusion (MCAO) modeling. They also used a fluorescent wide-field microscopic system and IR820 (a small-molecule organic dye) to perform high-contrast and large-penetration NIR-II fluorescent cerebral angiography, and obtained clear vascular changes before and after MCAO modeling in craniotomy mice. This study revealed that the highly biocompatible and bioexcretable IR-820 has great potential for therapeutic diagnosis in functional angiography. Based on the NIR II-MS *in vivo* microscopy system, the researchers also used a clinically-approved dye, Indocyanine Green (ICG), in mice without craniotomy (Yu et al., 2019). The changes in cerebral vessels in mice after cerebral embolization were further observed using a NIR-II fluorescence wide field microscopy system. The high-resolution NIR-II fluorescent wide field microscopic cerebrovascular imaging based on craniotomy can diagnose cerebral thrombosis in an intact skull, which enhances the feasibility of clinical application. The exploration of real-time tracking of cerebral thrombotic ischemia in mice opens up a new direction for *in situ* exploration of the pathogenesis of brain diseases. Qian Jun et al. (Cai et al., 2020) also used ICG as a NIR-II fluorescent probe to develop a wide-field microscopic system with high temporal resolution, achieving cerebrovascular imaging with a NIR-II fluorescent wide-field microscopic in macaques for the first time, measuring the blood flow velocity and heartbeat cycle of macaques, and identifying capillaries with a diameter of 7.8 μm at a depth of 300 µm.

The detection and diagnosis of tumors and inflammatory lesions is still a great clinical challenge, and the NIR-II fluorescence wide field microscopy system can also be used for accurate detection of tumors. Bawendi et al. (Bruns et al., 2017) used near-infrared quantum dots to image glioblastoma multiforme in the brain of mice at a frame rate of 30 fps through a transparent cranial window; this was enabled by the high temporal resolution of the fluorescent wide-field NIR-II microscopy system. They also performed principal component analysis to deconvolve the time series of images to distinguish pre-labeled tumors, arterial vessels, and venous vessels, which will benefit observation of changes in tumor blood vessel networks. Zhang Fan et al. (He et al., 2021) further distinguished tumor stroma and blood vessels by double-channel fluorescence angiography. The NIR-II fluorescent probe CEAF-OMe was applied to the microscopic imaging of tumor *in vivo*, and ICG was used as a vascular contrast agent to obtain two-color fluorescence images. The fluorescence signal of CEAF-OMe in tumor cells was specifically activated and could be distinguished from the ICG signal in blood vessels. This method helps avoid the serious fluorescence contamination caused by bleeding during surgery, and enables the accurate removal of the tumor. Wang Qiangbin et al. (Zhan et al., 2021) used the NIR-II fluorescent wide-field microscopic system to realize cell imaging, verified the targeting ability of the nanoparticle probe APP-Ag_2_S-RGD to cancer cells, and achieved accurate tumor resection through fluorescence-guided cells. Benzhong Tang et al. (Fan et al., 2021) injected AIE nanoparticles TQ-BPN into mice with old tumors (4 weeks) and new tumors (2 weeks), and used the NIR-II fluorescent wide-field fiber system to identify tumors at different stages of growth. The results showed that AIE nanoparticles could spread from the endovascular to the extravasal tissues, producing brighter fluorescent regions. Therefore, with the advantages of deep penetration depth and real-time imaging, NIR-II fluorescence wide-field microscopy system can be used to clearly visualize the enhanced permeability and retention (EPR) effect of tumors *in situ*, which is also conducive to early tumor detection and metastasis research.

The NIR-II fluorescent wide-field microscopy system offers high temporal resolution to monitor dynamic biological processes, high spatial resolution to observe tiny biological structures, precise location of drug distribution, and deep imaging depth. In addition, compared with other microscopy systems, the system is easy to use and the cost involved is moderate. It therefore has widespread application *in vivo* research and clinical practice. From cerebrovascular systems to tumor blood vessels to inflammatory tissues and isolated cells and tissue sections, imaging has been realized in mice, rats, and macaques, which demonstrates the great potential of the imaging technology of the NIR-II fluorescence wide-field microscopy.

## Conclusion and perspectives

In summary, thanks to the increasing abundance of probes, continuous improvements in optical detectors, and the innovation and improvement of system imaging optical paths and circuits, NIR-II fluorescence microscopic imaging technology has benefited from continuous innovation and breakthroughs have been made in terms of greater imaging depth, better SBR, and higher spatial resolution and imaging speed. Material technology, such as carbon nanotubes, conjugated polymers, semiconductor quantum dots, organic nanoparticles, small molecular organic dyes, and rare earth doped nanomaterials, has been developed and applied successfully. In addition, the progress in chip preparation and cooling technology has enabled the development and mass production of detectors with high quantum efficiency, large pixel, low noise, and wide motion. All these advances indicate that the NIR-II fluorescence microscopic imaging technology has a bright future in basic research and translation into the clinic. As more biological applications related to the cerebrovascular system, tumor blood vessels, and organ tissues, and more complex biological models from rodents to primates are reported, it is foreseeable that even richer and more complex clinical applications will be developed and realized.

In the field of microscope technology, as we have discussed before, confocal microscopy and multiphoton microscopy remain the gold standard for fluorescence bioimaging *in vivo* in visible or near-infrared spectroscopy. After nearly half a century of development, confocal technology has matured. However, the point scanning properties of confocal microscopes limit imaging experiments of dynamic samples to small volumes or a series of fixed samples at various stages of the process. LSFM is based on the optimization of existing fluorescence microscopy techniques and can generate effective signals for biomedical imaging. The limitations come from hardware such as detection sensors, especially when combined with other advanced beam-forming strategies. Four-dimensional data sets with high spatiotemporal resolution can be obtained, making it a powerful tool for a wide range of fluorescence imaging applications. As for wide-field fluorescence technology, although its background suppression is inferior to that of confocal microscopy and LSFM, its requirements for samples and experimental are significantly lower, which is conducive to clinical application. Therefore, further improvement is needed in the application of *in vivo* bioimaging.

To date, all NIR emitters mentioned are in the preclinical stage, making it particularly important to accelerate the clinical transition research process. Optimization of existing emitters and the development of new emitters require both optical properties (such as low attenuation coefficient, high quantum yield, and long emission wavelength), as well as biological properties (such as low or non-toxicity, small molecules, rapid excretion, and good biocompatibility). The combination of NIR fluorophores and LSFM achieves excellent imaging performance with a penetration depth of nearly 3 mm and a spatial resolution of less than 10 μm. Multiphoton microscopes using near-infrared contrast agents can eliminate self-fluorescence background. Multicolor STED technology has also been developed by using near-infrared fluorophores. Therefore, the long-term goal will be to combine and integrate advanced near-infrared fluorescence with microscopy-based beam shaping. Another direction of development is the hybrid mode of fluorescence imaging. Photoacoustic (PA) imaging can extend the imaging depth to 5 cm. Because several NIR fluorophores mentioned above are also available for this new technique, achieving superior *in vivo* fluorescence bioimaging with excellent spatial and temporal resolution and deeper detection penetration will be a huge improvement.

Based on existing research, there are still some aspects of NIR-II fluorescence microscopic imaging technology need improving in future. First, some fluorescent reagents pose safety problems in terms of immune intake and biological self-clearance, resulting in potential long-term toxicity in biological applications. This affects the further clinical application of NIR-II fluorescence microscopy. Second, NIR-II fluorescence microscopic imaging technology could be combined with other imaging methods (such as photoacoustic imaging). Multi-modal imaging could utilize the advantages of different modes to overcome the disadvantages of a single mode, so as to decipher more biological information and improve diagnostic accuracy. It is foreseeable that with more research and clinical work, NIR-II fluorescence microscopic imaging technology will take the lead in further clinical applications such as cerebrovascular health assessment, early cancer screening, and *in vitro* tissue detection. Third, because of the influence of background defocus signal, the spatial resolution of NIR-II fluorescence wide-field microscopy is still not ideal, and the imaging effect is inferior to that of confocal microscopy at the same depth. In order to further suppress the background, given the strong absorption of water at 1450 nm that can effectively inhibit the background signal, the NIR-IIx band has the best imaging potential. However, the imaging reagents that can be used for NIR-IIx and clinical application are still to be developed. In conclusion, the optimization of the imaging optical path and the innovation of fluorescence probes will greatly promote the improvement of fluorescence microscopy imaging. The continuous improvements in imaging depth and SBR of *in vivo* imaging will drive the vigorous development of NIR-II fluorescence microscopy-based *in vivo* imaging systems and techniques.
